# Anti-Inflammatory Activity of Citric Acid-Treated Wheat Germ Extract in Lipopolysaccharide-Stimulated Macrophages

**DOI:** 10.3390/nu9070730

**Published:** 2017-07-10

**Authors:** Hee-Yeong Jeong, Yong-Seok Choi, Jae-Kang Lee, Beom-Joon Lee, Woo-Ki Kim, Hee Kang

**Affiliations:** 1 Graduate School of East-West Medical Science, Kyung Hee University, Yongin 17104, Korea; jhy9592@naver.com; 2 Sajo DongA One Co., Ltd., Dangjin 31703, Korea; knownet@sajo.co.kr (Y.-S.C.); macmen@sajo.co.kr (J.-K.L.); 3 Department of Internal Medicine, College of Korean Medicine, Kyung Hee University, Seoul 02447, Korea; franchisjun@khu.ac.kr; 4 Department of Food Science and Biotechnology, Kyung Hee University, Yongin 17104, Korea; kimw@khu.ac.kr

**Keywords:** wheat germ, citric acid, inflammation, macrophage

## Abstract

Until recently, fermentation was the only processing used to improve the functionality of wheat germ. The release of 2,6-dimethoxy-1,4-benzoquinone (DMBQ) from hydroquinone glycosides during the fermentation process is considered a marker of quality control. Here, we treated wheat germ extract with citric acid (CWG) to release DMBQ and examined the anti-inflammatory activity of this extract using a lipopolysaccharide-activated macrophage model. Treatment of wheat germ with citric acid resulted in detectable release of DMBQ but reduced total phenolic and total flavonoid contents compared with untreated wheat germ extract (UWG). CWG inhibited secretion of the pro-inflammatory cytokines tumor necrosis factor-α, interleukin (IL)-6, and IL-12 and the synthesis of cyclooxygenase-2, while UWG only decreased IL-12 production. CWG and UWG induced high levels of anti-inflammatory IL-10 and heme oxygenase-1. CWG specifically inhibited phosphorylation of NF-κB p65 and p38 kinase at 15 min after LPS stimulation. Our study showed that citric acid treatment enhanced the anti-inflammatory activity of wheat germ extract.

## 1. Introduction

Wheat germ accounts for about 3% of the wheat grain and, despite its abundance of nutrients, is removed during the milling process [1]. Wheat germ contains α-linolenic acid (0.53%), sulfur containing amino acids (1.2%) such as glutathione, fibers (17.7%) such as lignins, oligosaccharides, and phytic acid, minerals (2.51%), and bioactive compounds such as tocopherols (0.02%), carotenoids, B group vitamins (0.01%), phytosterols (0.43%), policosanols, betaine (0.85%), alkylresorcinol, and polyphenols such as flavonoids, lignans and ferulic acid [2]. The presence of unsaturated fatty acids and oxidative and hydrolytic enzymes in wheat germ leads to rancidity, which makes preservation difficult [1]. Wheat germ also contains antinutritive factors such as raffinose, which is metabolized by gas-producing bacteria, and phytic acid, which interferes with mineral absorption [3]. Animals fed wheat germ agglutinin in high concentration were reported to develop hyperplastic and hypertrophic growth of the small intestines, hypertrophic growth of the pancreas, and thymus atrophy [4]. Thus, processing is required to stabilize or increase the nutritional value of wheat germ.

Inflammation is a physiological protective process that removes harmful substances in cooperation with immune and vascular cells. However, since neighboring normal cells are simultaneously destroyed, inflammatory responses must be tightly controlled. Macrophages play a major role in triggering and maintaining the inflammatory response. These cells produce inflammatory enzymes such as inducible nitric oxide synthase (iNOS) and cyclooxygenase-2 (COX2) as well as soluble cytokines such as tumor necrosis factor (TNF)-α, interleukin (IL)-6, and IL-12 through the NF-κB and MAPK (p38, JNK, and ERK) signaling pathways [5,6]. Such inflammatory proteins destroy microbes, activate endothelial cells, recruit blood immune cells, and influence the differentiation of T helper cells that further amplify the response of macrophages. On the other hand, macrophages can limit this activation by inducing anti-inflammatory cytokine IL-10 and heme oxygenase (HO)-1 [7].

Until recently, fermentation was the only processing used to improve the functionality of wheat germ. Extracts of wheat germ fermented by *Saccharomyces cerevisiae* showed anti-arthritic, anti-cancer and immunostimulatory effects and are now commercially supplied as over-the-counter medical nutriments under the brand name Avemar [8,9,10,11,12]. The manufacturing process of fermented wheat germ extract is standardized to 2,6-dimethoxy-1,4-benzoquinone (DMBQ) (0.4 mg/g on dry matter basis) [13]. DMBQ and methoxy-1,4-benzoquinone (MBQ) are oxidized forms of methoxyhydroquinones which are linked to oligosaccharides via a beta-1,6-glycosidic bond. It is unclear whether DMBQ is responsible for the functional activities of fermented wheat germ extract. A review of the literature shows that components of the extract, other than DMBQ, exert anti-cancer and immunostimulatory activities [10,14,15].

Koh et al. demonstrated the release of methoxyhydroquinones from the water soluble fraction of wheat flour through acid or beta glucosidase hydrolysis [16]. According to their work, acid hydrolysis produced a higher yield of methoxyhydroquinone than enzymatic hydrolysis. The aim of this study is to process wheat germ with citric acid, which is a strong, edible acid used in food applications, and evaluate the anti-inflammatory properties of citric acid-treated wheat germ extract (CWG) and untreated wheat germ extract (UWG) using lipopolysaccharide (LPS)-stimulated macrophages.

## 2. Materials and Methods

### 2.1. Preparation of CWG

Wheat germ was supplied by Sajo DongA One Co., Ltd. (Dangjin, Korea). The percentage of citric acid, incubation temperature, and time of acid hydrolysis were optimized to produce the maximum release of DMBQ. Preliminary results showed that optimal DMBQ release occurred with 50 g of unground wheat germ in 250 mL of deionized water (DW) stirred with 5% (*w*/*w*) citric acid (Sigma, St. Louis, MO, USA) for 8 h in a 60 °C water bath. The solution was centrifuged at 2500× *g* for 15 min, and then the supernatant was freeze-dried using a vacuum freeze-dryer. UWG was prepared under the same conditions. Moisture and ash content were determined according to the Approved Methods of the American Association of Cereal Chemists [17]. The crude protein content of these extracts was determined with a Pecatordigestor auto 24 (Foss, Hilleroed, Denmark) and a Kjeltec 8400 Automatic Titrator (Foss, Hilleroed, Denmark) using the Kjeldahl method.

### 2.2. HPLC Analysis of DMBQ

CWG or UWG (2 g) was dissolved in DW and extracted by shaking three times with 25 mL chloroform. The chloroform layers were collected and dehydrated with Na_2_SO_4_. The solvent was evaporated to dryness at 40 °C. The residue was re-dissolved in 5 mL of eluent and filtered through a 0.45-μm filter. HPLC analysis was performed using a SpectraSystem (ThermoFisher Scientific, San Jose, CA, USA) equipped with Discovery Rp-amide C16 column (5 μm, 250 × 4.6 mm) and a UV detector operated at 290 nm. The mobile phase was 20% acetonitrile in 25 mM KH_2_PO_4_, pH 4.8, with the flow rate and sample injection volume fixed at 0.7 mL/min and 20 μL, respectively. DMBQ (TCI, Tokyo, Japan) dissolved in eluent was used as a reference to calibrate the standard curve and retention times.

### 2.3. Analysis of Total Phenolic and Flavonoid Contents

The total phenolic content was measured by Folin-Ciocalteu colorimetry. Briefly, 0.2 mL of the appropriately diluted extracts was mixed with 2.6 mL of DW, 0.2 mL of Folin-Ciocalteu’s reagent (Sigma, St. Louis, MO, USA), and 2 mL of 7% Na_2_CO_3_ solution. The mixture was incubated at room temperature for 90 min and absorbance was measured at 750 nm using a S-4100 spectrophotometer (SCINCO, Seoul, Korea). The concentration of total phenolics was calculated as gallic acid (Sigma) equivalent (GAE) per gram of extract. For quantitation of total flavonoid content, 0.5 mL of the extract was mixed with 3.2 mL of DW, and 0.15 mL of 5% NaNO_2_ was added to the mixture. At 5 min, 0.15 mL of 10% AlCl_3_ was added, followed by 1 mL of 1 M NaOH. Absorbance was measured at 510 nm. The concentration of total flavonoids was expressed as mg catechin (Sigma) equivalents (CE) per gram of extract.

### 2.4. Isolation of Mouse Peritoneal Macrophages

Seven-week-old male Balb/c mice were obtained from SamTaco (Osan, Korea) and housed in a temperature- and humidity-controlled pathogen-free animal facility with a 12-h light-dark cycle. Our animal protocol (KHUASP(SE)-15-012) was approved by the Kyung Hee University Institutional Animal Care and Use Committee, and mice were cared for according to the US National Research Council for the Care and Use of Laboratory Animals (1996) specifications. Mice were injected intraperitoneally with 2 mL of 3.5% sterile thioglycollate solution (BD, Sparks, MD, USA); 4 days later, mice were sacrificed by cervical dislocation, and cells were isolated by peritoneal lavage with cold DMEM (Hyclone, Logan, UT, USA). After centrifugation, cells were suspended in DMEM with 10% fetal bovine serum (FBS) (Hyclone) and 1% penicillin-streptomycin. Cells were plated overnight at 37 °C, and non-adherent cells were removed.

### 2.5. Cell Viability Assay

Cell viability was determined using the MTS (3-(4,5-dimethylthiazol-2-yl)-5-3(carboxymethoxyphenyl)-2-(4-sulfophenyl)-2H-tetrazolium) reduction method (Promega, Madison, WI, USA) based on the measurement of mitochondrial respiration in live cells. Attached cells (4 × 10^4^ cells/0.1 mL) in 96-well plates were treated with increasing concentrations of CWG or UWG. After 24 h, the cells were treated with 20 μL/well MTS reagent. Optical density was measured at 490 nm using an iMark microplate reader (Bio-Rad, Hercules, CA, USA).

### 2.6. Nitrite Analysis

For nitric oxide determination, the mouse macrophage cell line RAW264.7 purchased from the Korea Cell Line Bank (Seoul, Korea) was cultured in DMEM supplemented with 10% FBS and 1% penicillin-streptomycin. Cells (1 × 10^6^ cells/mL) in 6 well plates were stimulated with 100 ng/mL LPS for 24 h in the presence of UWG or CWG. Fifty microliters supernatant was incubated with an equal volume of Griess reagent (Sigma) for 15 min at room temperature. We measured the absorbance at 550 nm with the microplate reader. Sodium nitrite was used as a standard.

### 2.7. Cytokine Analysis

Cells (2 × 10^6^ cells/mL) in 6 well plates were stimulated with 100 ng/mL LPS plus 0.5 ng/mL recombinant IFN-γ (BD Pharmingen, San Diego, CA, USA) and CWG or UWG was added simultaneously. After 24 h, supernatant was collected and levels of TNF-α, IL-6, IL-10, and IL-12 were determined by enzyme-linked immunosorbent assay (ELISA) according to the manufacturer’s protocol (BD Pharmingen). Briefly, flat-bottomed 96-well plates were coated overnight at 4 °C with coating antibodies (Abs). The plates were blocked with blocking buffer consisting of 10% FBS in phosphate buffered saline (PBS) for 1 h at room temperature. The plates were washed with wash buffer (0.05% Tween 20 in PBS) and appropriately diluted samples or standards were added. The plates were incubated for 2 h at room temperature. The supernatant was discarded and the wells were washed with wash buffer. Detecting Abs plus Avidin-horse radish peroxidase was added and incubated for 1 h at room temperature. After washing, tetramethybenzidine substrate solution (BD Pharmingen) was added. The color was allowed to develop for 30 min in the dark before the reaction was quenched with 0.2M H_2_SO_4_. The plates were then read at 450–570 nm and the sample concentrations were determined from a standard curve.

### 2.8. Western Blot Analysis

For iNOS, COX2, and HO-1 determination, cells (2 × 10^6^ cells/mL) in 6 well plates were stimulated with 100 ng/mL LPS plus 0.5 ng/mL recombinant IFN-γ and CWG or UWG was added simultaneously. After 24 h, cells were collected for Western blot analysis. For signaling molecule analysis, cells (3 × 10^6^ cells/2 mL) were pretreated with CWG or UWG for 1 h and then stimulated with LPS for 15 min. We chose this time period based on our previous experiments.

Cells were rinsed in cold PBS and then lysed on ice in RIPA buffer (50 mM Tris-HCl, pH 7.5; 150 mM NaCl; 1 mM EDTA; 20 mM NaF; 0.5% NP-40; and 1% Triton X-100) containing phosphatase inhibitor cocktail (Sigma) and protease inhibitor cocktail (Sigma). After centrifugation at 13,000× *g* for 10 min, supernatants were collected. Protein concentrations were determined using the Bradford protein assay reagent (Bio-Rad). 20 μg of protein was loaded and separated on an 8% or 10% SDS-polyacrylamide gel and transferred to polyvinylidene fluoride membranes. The membranes were blocked with 5% skim milk in Tris-buffered saline with 0.1% Tween 20 (TBST) for 1 h. The membranes were incubated with iNOS, IκBα, heme oxygenase 1, tubulin, GAPDH (Santa Cruz Biotechnology, Santa Cruz, CA, USA), COX2 (Cayman, Ann Arbor, MI, USA), phospho-JNK, JNK, phospho-ERK1/2, ERK1/2, phospho-p38, p38, phospho-NFκB-p65, NFκB-p65 (Cell Signaling Technology, Beverly, MA, USA) diluted in 5% skim milk in TBST overnight at 4 °C. The blots were washed with TBST and incubated for 1 h with anti-rabbit horseradish peroxidase-conjugated antibodies. Immunoreactive bands were detected with EzWEstLumi plus (ATTO, Tokyo, Japan) and analyzed using an EZ-Capture MG (ATTO). The band of each protein was quantified using ImageJ software.

### 2.9. Statistical Analysis

Data were analyzed by Student’s *t* test or ANOVA followed by the LSD test using SPSS 22 software (IBM, Chicago, IL, USA). *P* values less than 0.05 were considered significant.

## 3. Results

### 3.1. Determination of DMBQ, Total Phenolics, and Total Flavonoids

The chemical composition of UWG and CWG is as follows: in UWG, the moisture 12.4 ± 0.2%, crude protein 33.6 ± 0.2% of dry matter (d.m.), and ash 6.48 ± 0.01% of d.m.; in CWG, moisture 12.2 ± 0.1%, crude protein 21.6 ± 0.1% of d.m., and ash 6.41 ± 0.01% of d.m. Since DMBQ is now used as a quality marker of fermented wheat germ extract, we determined the amount of DMBQ in the extracts. No DMBQ in UWG was detected under our experimental conditions, and citric acid treatment increased the concentration of DMBQ to 0.065 mg/g. This value was much lower than the 0.2 to 0.4 mg/g reported elsewhere for fermentation [14,15,18]. Total phenolic and total flavonoid contents in UWG were reduced by 36% and 49%, respectively, after citric acid treatment (Table 1).

### 3.2. Effects of CWG on Cytotoxicity of Peritoneal Mouse Macrophages

**Figure 1:**
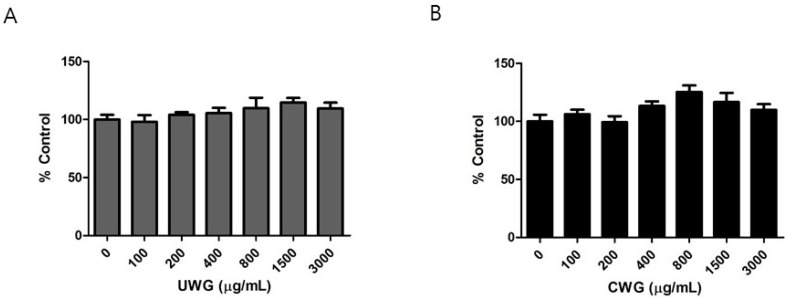
Effect of untreated wheat germ extract (UWG) and citric acid-treated wheat germ extract (CWG) on cell viability. Peritoneal macrophages isolated from Balb/c male mice were cultured with UWG (**A**) or CWG (**B**) for 24 h, and cell viability was determined using the MTS assay. Data are represented as percentage of control cells (0 μg/mL) (*n* = 4).

The cytotoxic response of CWG or UWG to peritoneal mouse macrophages was measured using the MTS assay. Extracts were added to cells for 24 h. The accumulation of formazan produced by various cellular dehydrogenases indirectly reflects the number of viable cells [19]. Doses up to 3000 μg/mL of CWG or UWG did not alter the amount of formazan, suggesting no toxic effects on cells (Figure 1). However, the MTT assay, the original version of the MTS assay, can overestimate the number of activated macrophages [20]. Macrophages cultured with polysaccharides, which stimulate these cells, may produce more formazan. Increasing concentrations of CWG or UWG contain a higher level of polysaccharides. Therefore, a large amount of formazan observed at high concentrations should be interpreted with caution. The peak plasma concentration after oral administration of Avemar is estimated to be 0.5–1 mg/mL [21]. Based on this, we used concentrations lower than 500 μg/mL for further assays.

### 3.3. Effects of CWG on iNOS, and COX-2 Expression in Macrophages Stimulated with LPS/IFN-γ

**Figure 2:**
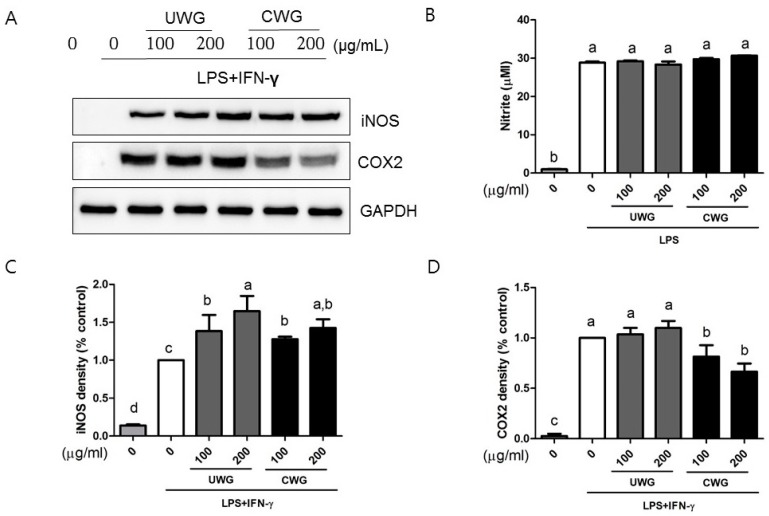
Effect of CWG on synthesis of inducible NO synthase (iNOS) and cyclooxygenase-2 (COX2). Mouse peritoneal macrophages were stimulated with LPS and interferon (IFN)-γ in the presence of UWG or CWG for 24 h. (**A**) The expression levels of iNOS and COX2 were analyzed by Western blotting using GAPDH as an internal control. One of three independent experiments is shown; (**B**) NO in the supernatant from LPS-stimulated RAW264.7 cells was measured by the Griess reaction (*n* = 2). RAW264.7 cells were stimulated with LPS in the presence of UWG or CWG for 24 h; (**C**,**D**) The band intensity of iNOS or COX2 was normalized with GAPDH and percent control (LPS/IFN-γ stimulated cells) was shown (*n* = 3). Different letters indicate statistical significances (*p* < 0.05).

Activated macrophages produce nitric oxide (NO), which is microbicidal and mediates the cytotoxic function of the cell. We first investigated whether CWG affects the expression of iNOS, the NO synthesizing enzyme. Because we isolated peritoneal macrophages from Balb/c mice, Th2 strains that exhibit a weak NO response, we used IFN-γ, a priming agent that potentiates the macrophage’s response to LPS [22]. Both UWG and CWG rather increased the expression of iNOS protein (Figure 2A,C). We also examined the response of the mouse macrophage cell line, RAW264.7, which does not require IFN-γ priming, and found a similar result. It is possible that NO generation could be altered without changes in level of iNOS protein. Since NO production varies in peritoneal macrophages from Balb/c mice, we examined NO in LPS stimulated RAW264.7 cells. The levels of nitrite was measured as an indicator of NO production. Increased iNOS activity induced by LPS was not prevented by CWG and UWG. Despite the increase in amount of iNOS protein, LPS-induced nitrite levels in cells treated with CWG or UWG did not increase (Figure 2B), indicating that iNOS enzymatic activity could be altered.

We examined another inflammatory protein, COX2 in peritoneal macrophages stimulated with IFN-γ and LPS. CWG, but not UWG, reduced COX2 synthesis (Figure 2A,D). Our results clearly showed that treatment of wheat germ with citric acid generates products that can decrease COX2 protein expression.

### 3.4. Effects of CWG on the Secretion of Proinflammatory Cytokines in LPS-Stimulated Macrophages

**Figure 3:**
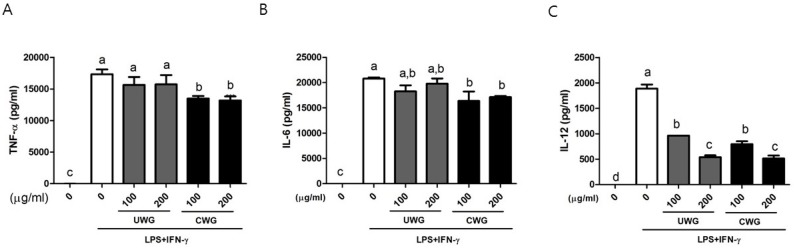
Effect of CWG on the secretion of tumor necrosis factor (TNF)-α, interleukin (IL)-6, and IL-12. Mouse peritoneal macrophages were stimulated with LPS plus IFN-γ in the presence of UWG or CWG for 24 h, and the levels of TNF-α (**A**), IL-6 (**B**), and IL-12 (**C**) in the supernatant were analyzed by ELISA. Data are expressed as mean ± SD (*n* = 3). Different letters indicate statistical significances (*p* < 0.05).

We further examined the levels of TNF-α, IL-6, and IL-12 in the supernatants collected from macrophages stimulated with LPS plus IFN-γ. CWG, but not UWG, decreased TNF-α secretion (Figure 3A). Both CWG and UWG reduced IL-6 secretion but statistical significance was observed only in CWG-treated cells compared with LPS/IFN-γ treated control cells (Figure 3B). These data showed that treatment of wheat germ with citric acid led to release of components that specifically target the secretion of TNF-α and IL-6, as well as COX2 synthesis. Both CWG and UWG reduced IL-12 secretion in a dose-dependent manner, with each equal potency and in a dose-dependent manner, indicating that citric acid did not contribute to the downregulation of IL-12 by UWG (Figure 3C).

### 3.5. Effects of CWG on Anti-Inflammatory Protein Expression in LPS-Stimulated Macrophages

**Figure 4:**
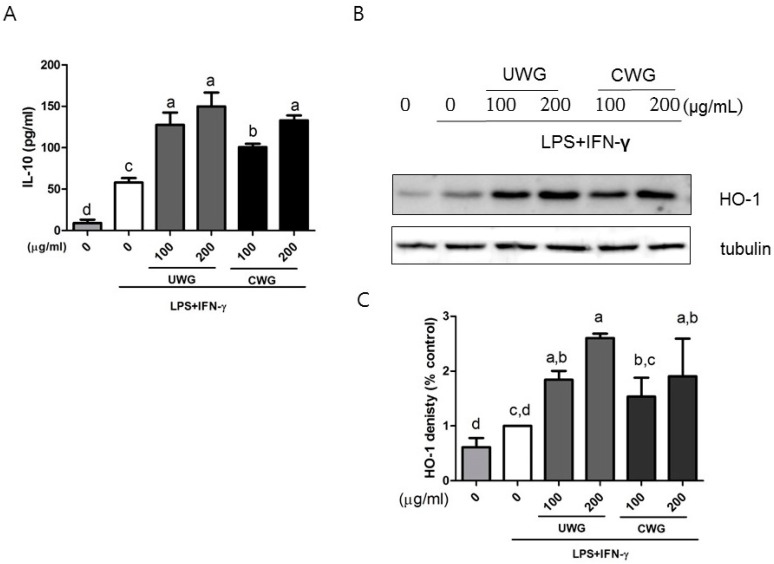
Effect of CWG on the secretion of IL-10 and the synthesis of heme oxygenase (HO)-1. Mouse peritoneal macrophages were stimulated with LPS plus IFN-γ in the presence of UWG or CWG for 24 h. (**A**) The level of IL-10 in the supernatant was analyzed by ELISA; (**B**) HO-1 synthesis was analyzed by Western blotting using tubulin as an internal control. One of three independent experiments is shown; (**C**) The band intensity of HO-1 was normalized with tubulin and percent control (LPS/IFN-γ stimulated cells) was shown. Data are expressed as mean ± SD (*n* = 3). Different letters indicate statistical significances (*p* < 0.05).

Macrophages produce IL-10 as a self-regulatory mechanism in response to LPS [23]. Both CWG and UWG induced more IL-10 secretion than treatment with LPS plus IFN-γ only (Figure 4A). HO-1 mediates part of the anti-inflammatory action of IL-10. We sought to determine whether CWG and UWG stimulate HO-1 expression. Both UWG and CWG induced expression of HO-1 (Figure 4B). The pattern of IL-10 and HO-1 by UWG or CWG was similar, suggesting that these two proteins are related to each other. These results indicate that components that induce IL-10 and HO-1 are present in wheat germ extract and that citric acid treatment does not add to this activity.

### 3.6. Effects of CWG on LPS-Induced NF-κB p65 and MAPK Activation

**Figure 5:**
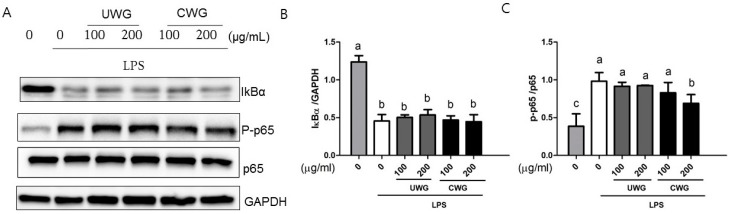
Effect of CWG on LPS-induced IκBα degradation and phosphorylation of NF-κB p65. Mouse peritoneal macrophages were pretreated with UWG or CWG for 1 h and then stimulated with LPS for 15 min. (**A**) The expression level of IκBα and phosphorylated p65 was determined by Western blotting. GAPDH was used as an internal control. One of three experiments is shown; (**B**,**C**) Bars represent the intensity of normalized target protein band (*n* = 3). Different letters indicate statistical significances (*p* < 0.05).

**Figure 6:**
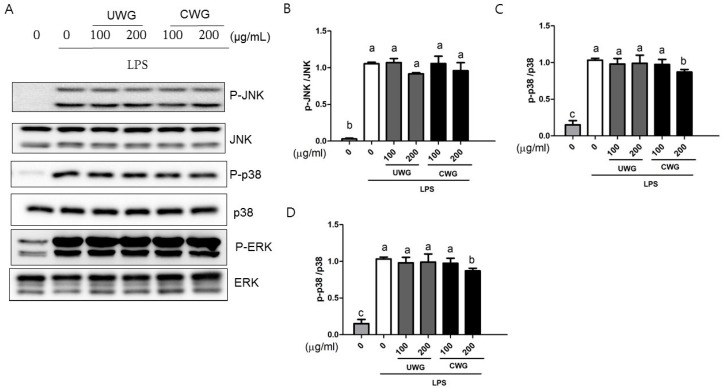
Effect of CWG on LPS-induced MAPK (JNK, ERK, and p38) activation. Mouse peritoneal macrophages were pretreated with UWG or CWG for 1 h and then stimulated with LPS for 15 min. (**A**) The expression level of phosphorylated MAPKs was determined by Western blotting. One of three experiments is shown; (**B**–**D**) Each phosphorylated protein was normalized with its total protein (*n* = 3). Different letters indicate statistical significances (*p* < 0.05).

The NF-κB and MAPK (p38, JNK, and ERK) pathways regulate the LPS-dependent transcriptional response that leads to expression of inflammatory proteins [6]. IκBα degradation is necessary for NF-κB activation [24]. Peritoneal macrophages were incubated with CWG or UWG 1 h prior to LPS stimulation. At 15 min after LPS stimulation, IκBα degradation and NF-κB p65 phosphorylation appeared (Figure 5). Neither CWG nor UWG affected IκBα degradation but NF-κB p65 phosphorylation was attenuated in cells treated with CWG at 200 μg/mL. We also analyzed MAPK activation at the same time points. We compared the phosphorylated amount of MAPK to its total amount. Among them, p38 phosphorylation declined in cells treated with CWG at 200 μg/mL (Figure 6). These data show that differences in the anti-inflammatory activity between UWG and CWG occur at least at the level of NF-κB and p38 activity.

## 4. Discussion

Whole grain is recommended for inclusion in diets because it has been reported to reduce markers associated with inflammation [25]. Whole grain consists of the endosperm, bran, and germ. Both the bran and germ are removed during the milling process, though they contain most of the antioxidant phytochemicals in wheat grain. In the present study, we showed that processing wheat germ with citric acid enhances the anti-inflammatory activity of wheat germ extract.

With regard to cytotoxicity, myeloid cell lines including the mouse macrophage cell line, RAW264.7 cell, were sensitive to the fermented wheat germ extract, Avemar, compared with non-myeloid cell lines: higher concentrations of Avemar (500 μg/mL and up) at 96 h inhibited cell viability [26]. In contrast, we found that the viability of mouse peritoneal macrophages was not affected at 24 h treatment with CWG and UWG at concentrations up to 3000 μg/mL. The same dose response applied to RAW264.7 cells. Although the culture duration was short, a lack of toxicity may be related to changes in constituents due to acid hydrolysis. As described above, the peak plasma concentration found following the ingestion of Avemar (9 g/day) is estimated to be 0.5–1.0 mg/mL. CWG at 100–200 μg/mL showed anti-inflammatory activity, suggesting that it may have therapeutic uses when given in such a pharmacological dose.

COX2 catalyzes the formation of prostaglandins and thromboxane from arachidonic acids, which is liberated from the membrane phospholipids by phospholipase A2 [27]. Aspirin and other non-steroidal anti-inflammatory drugs target COX2. Prostaglandin E_2_ is the most important product of the COX2 pathway and is responsible for the redness, heat, edema, and pain associated with inflammation. CWG, but not UWG, inhibited COX2 expression. Supplementation of fermented wheat germ extract to arthritic rats was shown to ameliorate inflammation symptoms and decrease COX2 gene expression in white blood cells [8]. It is not clear whether the substance released by citric acid treatment causes the same effect. We cannot exclude the possibility that UWG in higher concentrations might exert the same activity.

TNF-α, IL-6, and IL-12 are representative inflammatory cytokines produced by LPS-stimulated macrophages while IL-10 can antagonize the action of these inflammatory cytokines. TNF-α and IL-6 were decreased by citric acid treatment, but IL-12 and IL-10 were both targets shared by CWG and UWG. Based on these results, citric acid treatment releases unidentified components in wheat germ that specifically interfere with TNF-α and IL-6, as well as COX-2, but it did not influence on IL-12 and IL-10.

The NF-κB pathway rapidly induces the genes that have important roles in inflammation. Under resting conditions, NF-κB is held by IκB in the cytosol and upon various inflammatory stimuli, IκB degradation occurs and free NF-κB migrates to the nucleus where phosphorylated NF-κB dimers bind to κB DNA elements and induce gene transcription [28]. NF-κB consists of p65 (Rel A), Rel B, c-Rel, p50, and p52, and exists as homo/hetero dimers. Among these components, p65, Rel B, and c-Rel have the transcription activation domain necessary for the upregulation of genes, and p65:p50 is the primary target of IκBα [29]. Regulation of the NF-κB pathway can be achieved in a number of ways. We did not observe a marked effect in IκBα degradation in cells treated with CWG or UWG but found a reduced phosphorylation of NF-κB p65 in CWG treatment. We also found p38 attenuation in CWG-treated cells. P38 kinase is involved in LPS-stimulated TNF-α and IL-6 production by directly modulating these cytokine mRNA half-life or modulating cytosolic phospholipase A2 -mediated NF-κB activation [30,31]. These events may account for the observed difference in the anti-inflammatory properties of CWG and UWG.

The binding of IL-10 to its receptor induces production of HO-1. Additionally, HO-1 deficiency blocks the anti-inflammatory action of IL-10 in a sepsis model [7]. HO-1 is a stress-inducible protein generated by various stimuli such as heavy metals, UV light, oxidative stress, and certain cytokines [32]. The byproducts of HO-1 enzymatic activity through oxidative degradation of heme are carbon monoxide, ferrous irons, and biliverdin/bilirubin [32]. Among these products, carbon monoxide is directly involved in the inhibitory action of HO-1 against TNF-α and iNOS [7]. CWG and UWG increased iNOS protein synthesis in activated macrophages, but not NO generation. This may be explained by the increased cytoprotective function of HO-1.

Studies related to the biological effects of processed wheat germ have used DMBQ as a quality marker. DMBQ is believed to have anticancer and antibacterial activities [33,34]. DMBQ was not detectable from unground wheat germ treated under our experimental conditions of shaking in DW at 60 °C. Our preliminary study showed that under different conditions in which wheat germ was incubated in DW at 30 °C, 0.190 mg/g DMBQ were found. Various microorganisms such as yeasts and lactic acid bacteria are found in wheat germ [3]. The microbial beta glucosidase that exists in wheat germ might exert its activity under optimum conditions.

A study on fermented wheat germ extract showed that components other than DMBQ have the immunostimulatory action of fermented wheat germ in mice [10]. However, this study did not include animals fed raw wheat germ extract as a comparison, which made it unclear that such effects were derived from fermentation. Our study shows that UWG itself has anti-inflammatory activity, albeit less potent activity than CWG. DMBQ is the least likely to be responsible for the activity of UWG. We did not clarify the detailed change in composition following citric acid treatment. We only noted reductions in total phenolics and total flavonoid content. These results align with a previous report that production of DMBQ and MBQ are inversely correlated with that of flavonoids during wheat germ fermentation [35]. Numerous studies demonstrate the anti-inflammatory activity of flavonoids [36]. Our results suggest that components other than flavonoids contribute to the enhanced anti-inflammatory activity of CWG. A bioactivity-guided fractionation study of UWG or CWG is required to elucidate their active components.

Many nutritionally valuable components in wheat germ are embedded in a complex matrix. The choice of an appropriate extraction method has to be determined according to the yield of the target molecules. When it comes to DMBQ only, acid hydrolysis was more effective in yielding the release of DMBQ precursors from wheat and less time consuming than enzymatic hydrolysis [16]. Up to now, most reports on the biological functions of wheat germ are based on fermentation, which requires microbial enzymatic reaction. Generally, fermentation or enzymatic hydrolysis is substrate-specific, producing desirable by-products but costly and time-consuming while acid hydrolysis is fast and economical but producing undesirable by-products due to random breakage of glycosidic bonds [37]. However, the efficacy of different treatments needs to be evaluated.

## 5. Conclusions

Here, we demonstrated that wheat germ extract showed anti-inflammatory activity, and treatment of wheat germ with citric acid enhanced this function in an LPS-stimulated macrophage model. In vivo studies are required to validate the activity of wheat germ extract.

## Figures and Tables

**Table 1:** Contents of total phenolics and flavonoids and DMBQ.

	UWG	CWG
Total phenolics (mg GAE/g)	15.77 ± 0.03 ^a^	10.14 ± 0.07 ^b^
Total flavonoids (mg CE/g)	13.69 ± 0.13 ^a^	7.02 ± 0.07 ^b^
DMBQ (mg/g)	Not detected	0.065 ± 0.003

UWG: untreated wheat germ extract; CWG: citric acid-treated wheat germ extract. Values with different letters in one row are significantly different (*p* < 0.05). Data represent mean ± SD (*n* = 3).
